# Dysregulation of hypoxia-inducible factor 1α in the sympathetic nervous system accelerates diabetic cardiomyopathy

**DOI:** 10.1186/s12933-023-01824-5

**Published:** 2023-04-18

**Authors:** Petra Hrabalova, Romana Bohuslavova, Katerina Matejkova, Frantisek Papousek, David Sedmera, Pavel Abaffy, Frantisek Kolar, Gabriela Pavlinkova

**Affiliations:** 1 Laboratory of Molecular Pathogenetics, Institute of Biotechnology CAS, BIOCEV, Vestec, Czechia; 2 Charles University, Prague, Czechia; 3 Institute of Physiology CAS, Prague, Czechia; 4 Institute of Anatomy, Charles University, Prague, Czechia; 5 Laboratory of Gene Expression, Institute of Biotechnology CAS, BIOCEV, Vestec, Czechia

**Keywords:** Cardiac function, Inflammation, Diabetic cardiomyopathy, Collagen deposition, Sympathetic neurons

## Abstract

**Background:**

An altered sympathetic nervous system is implicated in many cardiac pathologies, ranging from sudden infant death syndrome to common diseases of adulthood such as hypertension, myocardial ischemia, cardiac arrhythmias, myocardial infarction, and heart failure. Although the mechanisms responsible for disruption of this well-organized system are the subject of intensive investigations, the exact processes controlling the cardiac sympathetic nervous system are still not fully understood. A conditional knockout of the *Hif1a* gene was reported to affect the development of sympathetic ganglia and sympathetic innervation of the heart. This study characterized how the combination of HIF-1α deficiency and streptozotocin (STZ)-induced diabetes affects the cardiac sympathetic nervous system and heart function of adult animals.

**Methods:**

Molecular characteristics of *Hif1a* deficient sympathetic neurons were identified by RNA sequencing. Diabetes was induced in *Hif1a* knockout and control mice by low doses of STZ treatment. Heart function was assessed by echocardiography. Mechanisms involved in adverse structural remodeling of the myocardium, i.e. advanced glycation end products, fibrosis, cell death, and inflammation, was assessed by immunohistological analyses.

**Results:**

We demonstrated that the deletion of *Hif1a* alters the transcriptome of sympathetic neurons, and that diabetic mice with the *Hif1a*-deficient sympathetic system have significant systolic dysfunction, worsened cardiac sympathetic innervation, and structural remodeling of the myocardium.

**Conclusions:**

We provide evidence that the combination of diabetes and the *Hif1a* deficient sympathetic nervous system results in compromised cardiac performance and accelerated adverse myocardial remodeling, associated with the progression of diabetic cardiomyopathy.

**Supplementary Information:**

The online version contains supplementary material available at 10.1186/s12933-023-01824-5.

## Background

Heart function is tightly controlled by the autonomic nervous system, which is comprised of parasympathetic and sympathetic neurons. The cardiac sympathetic nervous system uses the catecholamine norepinephrine as a neurotransmitter, and modulates heart rate, conduction velocity, myocardial contractility, cardiomyocyte size and structure [1–3]. The sympathetic nervous system is composed of preganglionic neurons, residing in the spinal cord forming the preganglionic motor column, and of postganglionic neurons, forming the sympathetic ganglion chain [1]. Postganglionic sympathetic neurons innervating the heart are located primarily in the stellate ganglion, with minor contributions from the middle cervical and upper thoracic paravertebral sympathetic ganglia [4, 5]. Cardiac sympathetic neurons extend axons across the epicardium along large-diameter coronary vessels into the myocardium [6]. These axons branch out and form varicosities along their length with synaptic vesicles that contain norepinephrine. Cardiac sympathetic innervation is most dense in the central conduction system, including the His bundle, and the sinoatrial and atrioventricular nodes, and in the subepicardium [1]. The sympathetic innervation density gradually decreases from the atria to the ventricles and from the base to the apex of the heart. These regional differences in the sympathetic innervation pattern are necessary to effectively control heart performance. Changes in the innervation pattern and activity of the sympathetic nervous system are implicated in many cardiac pathologies, including hypertension, myocardial ischemia, cardiac arrhythmias, sudden cardiac death, and heart failure [7].

Compromised cardiac performance, sympathetic nervous system abnormalities, and aberrant myocardial remodeling are hallmarks of diabetic cardiomyopathy (as reviewed in [8–10]). Diabetic cardiomyopathy is initially characterized by myocardial fibrosis, cardiomyocyte stiffness, and diastolic dysfunction that then progresses to systolic dysfunction that often evolves to heart failure [8, 11]. Underlying molecular factors include increased production of advanced glycation end products (AGEs), tissue inflammation, oxidative stress and mitochondrial dysfunction, changes in cellular metabolism, and accumulation of collagens. The cardiac sympathetic nervous system of the diabetic heart is initially hyperactive with elevated whole-body norepinephrine spillover, but later sympathetic innervation is reduced together with decreased nerve growth factor production by cardiomyocytes [12–14].

The transcription factor HIF-1 is required for physiological responses to hypoxia and plays a critical role in the pathophysiology of human diseases, such as cancer, cardiovascular disease, and diabetes [15, 16]. The haploinsufficiency of the α subunit (*Hif1a*), whose regulation is oxygen dependent, results in impaired responses to hypoxia and ischemia in mouse models [17–20]. Although diabetes is associated with increased tissue hypoxia, optimal activation of HIF-1α is blunted in the diabetic environment, affecting the adaptive hypoxic responses [21–25], reviewed in [26, 27]. Correspondingly, the combination of *Hif1a* haploinsufficiency and diabetes accelerated the pathological changes in the heart, such as the functional impairment of the left ventricle accompanied by cardiac remodeling [28], and in combination with an adverse diabetic environment in utero increased susceptibility to congenital heart defects and to cardiac dysfunction [29, 30].

HIF-2α (also known as EPAS1) is another closely related isoform, which also activates HRE-dependent gene transcription. HIF-1α and HIF-2α are essential for acute oxygen sensing and their coordinated transcriptional activation is critical for physiological adaptations to hypoxia, including increase in respiration and cardiac output, reviewed in [31]. HIF-1α or HIF-2α tissue-specific and temporal expression profiles are distinct, as there are the phenotypes of *Hif1a*^*–/–*^ and *Hif2a*^*–/–*^ mice, suggesting some functionally nonredundant roles [32–35]. A conditional deletion of HIF-2α in sympathoadrenal lineages affects survival of neuroendocrine glomus cells of the carotid body after birth without affecting chromaffin cells or sympathetic neurons [36]. In adults, the neuroendocrine glomus cells of the carotid body serve as primary regulators of sympathetic nerve activity. This regulation is mediated by HIF-1α, which activates the glomus cells, while HIF-2α opposes its action [37]. Opposing roles of HIF-1α and HIF-2α have been also shown in proliferation and differentiation of catecholamine-producing tumors (pheochromocytomas and paragangliomas) [38]. High expression of *HIF2a* results in a proliferative immature phenotype, whereas high expression of *HIF1a* leads to mature catecholamine phenotypic features. Thus, the requirements for HIF-1α and HIF-2α in the development and maturation of glomus cells, chromaffin cells, and sympathetic neurons are still ambiguous. Unlike HIF-1α, there are relatively a few papers reporting a role of HIF-2α in insulin signaling and diabetes [39–41].

We have previously revealed a key role for HIF-1α in the development of sympathetic neurons and sympathetic innervation of the heart [42]. To bypass the early embryonic lethality associated with germline *Hif1a* deletion [33, 43], we generated conditional deletion of a floxed *Hif1a* [44] using *Isl1*^*Cre*^ (*Hif1aCKO*). Previously, we found that *Hif1a* deletion impaired the survival and proliferation of preganglionic and postganglionic neurons of the sympathetic nervous system, survival of neuroendocrine chromaffin cells in the adrenal gland, the expression of tyrosine hydroxylase (TH), a direct target of HIF-1, and sympathetic innervation of *Hif1aCKO* hearts, without affecting heart development [42]. These findings indicate that the *Hif1a*-deficient sympathoadrenal cell lineage may also play a role in various sympathetic abnormalities underlying cardiac pathologies, such as sudden cardiac death and heart failure. Therefore, we questioned whether the *Hif1a-*deficient sympathetic nervous system would affect the development of diabetic cardiomyopathy. We hypothesized that an early phase of diabetes-exposure would be the best option to uncover the effects of the *Hif1a*-deficient sympathetic system on functional and structural adaptations of the diabetic heart, and consequently, any acceleration of the early-phase pathological effects of diabetes on the heart. In the present study, using the same model, we report that *Hif1a* deficiency in sympathetic neurons in combination with diabetes resulted in worsened cardiac function, accelerated adverse structural remodeling and sympathetic denervation in the heart.

## Methods

### Experimental animals

This study was approved by the local Animal Care and Use Committee of the Institute of Molecular Genetics. All experiments were performed using 4 months old males. Animals were housed in a controlled environment with 12 h light/dark cycles and free access to water and food. For obtaining the mouse model with conditional deletion of *Hif1a* (*Hif1aCKO*) we crossbred *Isl1*^*Cre/*+^ knock-in mice (Isl1^tm1(cre)Sev^/^+^) [45] with mice carrying floxed *Hif1a*^*tm3Rsjo*^ with exon 2 of *Hif1a* gene flanked by loxP sites (*Hif1a*^*loxP/loxP*^) [44]. *Isl1*-*Cre*^−/−^*; Hif1a*^*loxP/*+^ or *Isl1*-*Cre*^*−/−*^*;Hif1a*^*loxP/loxP*^ mice were used as controls. Control and heterozygous *Isl1*-*Cre*; *Hif1a*^*loxP/*+^ mice were born and survived according to the Mendelian ratio of genotypes. We also used *tdTomatoAi14* reporter mice with *Rosa-CAG-LSL-tdTomato* allele (Ai14, B6.Cg-*Gt(ROSA)26Sor*^*tm14(CAG−tdTomato)Hze*^, Stock No: 7914 Jackson Laboratory). The reporter *Hif1aCKO*-*Ai14* (genotype: *Hif1a*^*loxP/loxP*^*; Isl1*^*Cre*^*; tdTomatoAi14*) and control-*Ai14* mice (genotype: *Hif1a*^*loxP/*+^*; Isl1*^*Cre*^*; tdTomatoAi14*) were used in our molecular and phenotypic analyses. All comparisons were made between animals with the same genetic background. Phenotyping and data analysis were performed blind to the genotype of the mice (primer sequences for genotyping are listed in Additional file 1: Table S1).

### Diabetes induction

Diabetes was induced in males at 6 weeks of age by 2 intraperitoneal injections of 100 mg/kg body weight of streptozotocin (STZ, S0130, Sigma-Aldrich, USA) within a one-week interval, as described [28, 29]. The level of blood glucose was checked in a drop of blood from the tail vessel using a glucometer (COUNTOUR plus ONE, Ascensia Diabetes Care, Switzerland). Mice with level of glucose maintained above 13.9 mmol/l in blood were classified as diabetic. After 2 months from the induction of diabetes, diabetic males and non-diabetic control males (4 months of age) were killed, and their tissues were collected for analyses.

### Echocardiography

The echocardiographic evaluation of the geometrical and functional parameters of the left ventricle (LV) was performed using the GE Vivid 7 Dimension (GE Vingmed Ultrasound, Horten, Norway) with a 12 MHz linear matrix probe M12L. The diastolic and systolic dimensions of the LV including anterior and posterior wall thickness (AWTd, PWTd, AWTs, PWTs) and cavity diameter (LVDd, LVDs) were measured in the 4-month-old animals as previously described [17, 42]. The following functional parameters were derived from these dimensions: fractional shortening (FS) = 100 * [(LVDd-LVDs)/LVDd] and ejection fraction (EF) = 100 * (LVDd^3^ − LVDs^3^)/LVDd^3^. Diastolic and systolic cavity volumes (EDV, ESV) were calculated based on prolate spheroid geometry using the formula EDV = 0.001 * (4 * π/3) * k * LVDd^3^/8 and ESV = 0.001 * (4 * π/3) * k * LVDs^3^/8, where (k) is a ratio of long to short axis. Stroke volume (SV) = EDV–ESV, cardiac output (CO) = SV * HR, where (HR) is heart rate, and cardiac index (CI) = CO/BW, where (BW) is the body weight.

### RNA sequencing of fluorescence-activated cell sorted sympathetic ganglion neurons

Sympathetic ganglia with *tdTomatoAi14* fluorescent reporter were dissected from male mice. Sympathetic chains were homogenized in Hanks’ Balance Salt Solution (H6648, Sigma-Aldrich, USA), neuronal cells were dissociated using 0.1% collagenase for 5 min (C9263, Sigma-Aldrich, USA), then cells were treated by 0.25% trypsin in Dulbecco’s PBS for 7 min (T4799, Sigma-Aldrich, USA), and cells were washed between treatments using Dulbecco’s PBS (D5662, Sigma-Aldrich, USA) based on the protocol [46]. Enzymatic activity was stopped by adding FACS buffer (2% FBS in Dulbecco’s PBS and 10 mM EGTA). Fluorescence-activated cell sorting (FACS) of tdTomato positive cells was performed at the Imaging Methods Core Facility at BIOCEV on a BD FACS Aria Fusion flow cytometer operated with BD FACSDiva™ Software (gating strategy in Additional file 1: Fig. S1). 100 tdTomato^+^ cells from one sympathetic ganglion chain were collected into individual wells of 96-well plate containing 5 µl of lysis buffer of NEB Next single-cell low input RNA library prep kit for Illumina (New England Biolabs #E6420, USA). Plates were frozen immediately on dry ice and stored at − 80 °C. The total time from euthanasia to cell collection was ∼ 3 h.

RNA-seq libraries were prepared from *Hif1aCKO-Ai14* mutant (n = 5) and control-*Ai14* (n = 3). Each sample contained 100 tdTomato^+^ sympathetic neurons. Following the manufacturer’s instructions, the NEB Next single-cell low input RNA library prep kit for Illumina was used for cDNA synthesis, amplification, and library generation [47] at the Gene Core Facility (Institute of Biotechnology CAS, Czechia). Fragment Analyzer assessed the quality of cDNA libraries. The libraries were sequenced on an Illumina NextSeq 500 next-generation sequencer. NextSeq 500/550 High Output kit 75 cycles (Illumina #200024906) were processed at the Genomics and Bioinformatics Core Facility (Institute of Molecular Genetics CAS, Czechia). RNA-Seq reads in FASTQ files were mapped to the mouse genome using STAR (version 2.7.0c [48]). GRCm38 primary assembly and annotation version M8. The raw data of RNA sequencing were processed with a standard pipeline. Using cutadapt v1.18 [49], the number of reads (minimum, 32 million; maximum, 73 million) was trimmed by Illumina sequencing adaptor and of bases with reading quality lower than 20, subsequently reads shorter than 20 bp were filtered out. TrimmomaticPE version 0.36 was used for quality control tasks [50]. Ribosomal RNA and reads mapping to UniVec database were filtered out using bowtie v1.2.2. with parameters -S -n 1 and SortMeRNA [51]. A count table was generated by Rsubread v2.0.1 package using default parameters without counting multi mapping reads. The raw RNA-seq data were deposited at GEO: (https://www.ncbi.nlm.nih.gov/geo/).

DESeq2 (v1.26.0 [52]) default parameters were used to normalize data and compare the different groups. Differentially expressed genes were identified based on an adjusted P-value p_adj_ < 0.01, FC > 1.5, and a base mean ≥ 50 was applied to identify differentially expressed genes between *Hif1aCKO* mutant and control neurons. The volcano plot was constructed using programming language R (packages ggplot2 version 3. 3. 6. and ggrepel 0.9.1.). The functional annotation of the differentially expressed genes was performed using GOTermFinder and g: Profiler. Complete query details are available in Query info tabs in Additional file 10: Dataset S2. The resulting GEM and combined GMT files were loaded into Cytoscape [53] plugin “EnrichmentMap” [54] using 0.01 FDR q-value cutoff to generate a network. The edge cutoff was set to 0.55, and nodes were filtered by gs_size < 80. In the Enrichment map of upregulated genes, separate nodes sharing the same function (e. g. tube development and epithelial tube development) or having very general function (e. g. regulation of signal transduction) were removed. In the Enrichment map of downregulated genes, the cluster depicting cellular interactions was removed. Further adjustments were made in yFiles Layout Algorithms, Legend Creator (Cytoscape plugins).

### Quantitative real-time PCR

RNA was isolated from dissected sympathetic chains of individual adult males using Trizol (Invitrogen, USA). The concentration and purity were quantified using NanoDrop (ND-2000 Spectrophotometers, Thermo Fisher Scientific, USA). cDNA samples were prepared using Maxima H Minus First Strand cDNA Synthesis Kit with dsDNA (#K1682, Thermo Scientific, USA) from RNA collected samples (total RNA 0.25 µg). Quantitative real-time PCR (qRT-PCR) was performed with 10 × diluted cDNA samples. 4 µl cDNA was added to 5 µl of SybrGreen (GrandMaster Mix, TATAA Biocenter, Sweden) with 0.2 µM reverse and forward primers. Primers were designed using Primer 3 software and sequences are in Additional file 1: Table S2. Validation of RNA-seq targets were performed by Bio-Rad C1000 Thermal Cycler (CFX384 Real-Time system, USA), activation AmpliTaq at 95 °C for 10 min, followed by 40 cycles at 95 °C for 15 s for denaturation, and 60 °C for 60 s for extension. The relative expression levels of mRNA of target genes were normalized to the reference gene *Hprt1*. All reactions were conducted in duplicates and the data were calculated using the ΔΔCp method, as previously described [29, 55].

### Western blot assays

The diabetic and non-diabetic hearts, and adrenal glands were homogenized with RIPA (Radio Immuno Precipitation Assay buffer) containing phosphatase and protease inhibitors, as described [28, 29]. Briefly, protein lysates (total protein 50 µg or 100 µg) were resolved using 6%, 8% or 10% SDS-PAGE gels. The membranes were blocked (5% dry milk) and overnight incubated with anti-TH (tyrosine hydroxylase), anti-CHAT (choline acetyltransferase), anti-COL1 (collagen type I), anti-chromogranin A (ChgA), anti-HIF2α, and anti-NGF (nerve growth factor) antibodies (Additional file 1: Table S3). The secondary antibodies were horseradish peroxidase conjugated IgG (Sigma-Aldrich, USA) at 1:10,000 in 1% dry milk (Additional file 1: Table S4). Membranes were developed using the SuperSignal West Femto maximum Sensitivity Substrate (#34095, Thermo Fisher Scientific, USA). Restore Plus Western Blot Stripping Buffer (#46430, Thermo Fisher Scientific, USA) was used to remove bound antibodies, and after that Membrane Fraction WB Coctail (#ab140365, Abcam, UK) or beta actin (#5125, Cell Signaling, USA) were used. The chemiluminescent signals were detected by ImageQuant LAS 4000 Imager (GE Healthcare-Bio-Sciences AB, USA) and analyzed by the gel quantification tool NIH ImageJ software [56].

### Immunohistochemistry and morphological evaluations

Whole-mount immunohistochemical staining of adult hearts were performed on cleared tissue, as described [57]. Adult hearts were dissected and perfused with 0.05% heparin in PBS, and 4% paraformaldehyde (PFA) and fixed in 4% PFA for hour. The tissue was cleared with CUBIC reagent at 37 °C with gentle shaking for 1 month. Anti-TH antibody was used for the visualization of sympathetic innervation. Vibratome sections (adrenal gland and stellate ganglion 80 µm, and heart 100 µm thick) were stained with anti-WT1 (Wilms´ tumor-1), anti-F4/80, anti-NeuN, and anti-TH antibodies (Additional file 1: Table S3). The nuclei were stained with Hoechst 33258 (Sigma-Aldrich, USA). The secondary antibodies were Alexa Fluor 488, 594, 647 conjugated (Additional file 1: Table S4). The fluorescent signals were detected by LSM 880 NLO (Carl Zeiss AxioObserver.Z1, Germany) and AxioZoomV16 (Carl Zeiss, Germany). Paraffin sections (8 µm) were stained with Picrosirius Red (collagen staining, ab244887, Abcam, UK) Periodic acid-Schiff [PAS, staining of advanced glycation and products (AGEs), 395B-1KT, Sigma-Aldrich, USA] and TUNEL (#11684795910, Sigma-Aldrich, USA). ImageJ and ZEN software were used for image processing. The “Threshold” function ImageJ was used for the quantification of TH^+^, PAS^+^, and picrosirius red^+^ areas, and expressed as a percentage of analyzed total area. Positive WT1, NeuN, and F4/80 cells were counted using the “Cell counter” function ImageJ. TUNEL^+^ cells were counted in three consecutive transversal paraffin sections of the LV.

### Quantification of epinephrine by competitive enzyme immunoassay

The Enzyme-Linked Immunosorbent Assay (ELISA) was performed to measure epinephrine levels in plasma samples obtained from 4 months old diabetic and non-diabetic control and mutant male mice. Blood was collected from the facial vein directly to anticoagulant EDTA-treated tubes. Cells were removed from plasma by centrifugation for 15 min at 1500 ×*g* at 4 °C. Subsequently, plasma was collected and quickly frozen at − 80 °C. Quantitative detection of epinephrine was performed using competitive EIA (enzyme immunoassay) kit (#LS-F55506, LSBio, USA) in 96-Well Strip Plate with the lowest detection limit 2.54 picograms/mL. The optical density of each sample was performed at 450 nm using high performance plate reader CLARIOStar (BMG Labtech, Germany). Concentration of each sample was calculated from the linear equation of standard using GraphPad Prism 9.4.1.

### Light-sheet fluorescent microscopy (LFSM) and analysis of images

The secondary sympathetic chain was microdissected from non-diabetic and diabetic, control-*Ai14* and *Hif1aCKO-Ai14* mice (4 months-of age). We used an advanced CUBIC protocol [57] for tissue clearing to enable efficient imaging by light-sheet microscopy. Samples were stored before imaging in Cubic 2 at room temperature. Zeiss Lightsheet Z.1 microscope (Carl Zeiss, Germany) with illumination objective Lightsheet Z.1 5x/0.1 and detection objective Dry objective Lightsheet Z.1 5x/0.16 was used for imaging at the Light Microscopy Core Facility of the Institute of Molecular Genetics, CAS, Czechia. IMARIS software v8.1.1 (Bitplane AG, CA, USA) was used for image processing.

### Statistical analysis

Statistical analyses were performed using GraphPad Prism 9.4.1, results are considered significant at P values of < 0.05. Graphs showed only P values less than or equal to 0.05 in multiple comparison tests. Data sets with two groups (non-diabetic vs. diabetic) and two genotypes (control vs. *Hif1aCKO*) were analyze with two-way ANOVA testing differences among experimental groups based on the genotype and experimental condition (diabetic exposure or non-diabetic exposure) followed by post hoc comparisons tests. Data are expressed as mean ± SD or mean ± SEM. Sample sizes and individual statistical results for all analyses are provided in the figure legends and tables.

## Results

### Cardiac performance is impaired in diabetic *Hif1aCKO* mice

**Fig. 1:**
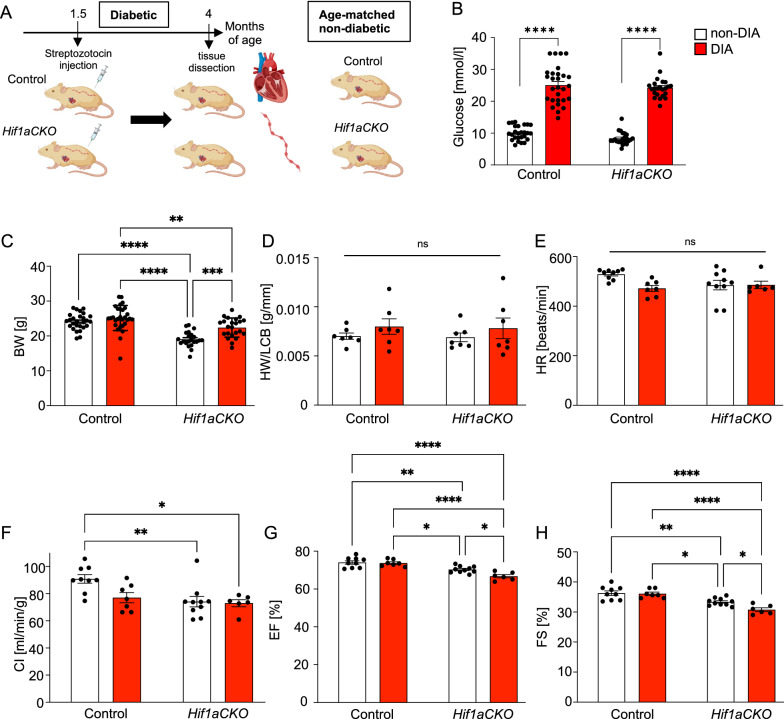
Heart function affected by the combination of diabetes and the *Hif1a* deficient sympathetic system. **A** Experimental design. **B** Blood glucose levels and **C** body weight (BW) (non-DIA Control n = 27, DIA Control n = 28, non-DIA *Hif1aCKO* n = 22, DIA *Hif1aCKO* n = 23), and **D** the heart weight adjusted to the length of the cranial base (HW/LCB; n = 7) measurements of 4-month-old non-diabetic and diabetic, control and *Hif1aCKO* mice. **E–H** The echocardiographic evaluation of left ventricular systolic function and hemodynamics: heart rate (HR), cardiac index (CI), ejection fraction (EF), and fractional shortening (FS) (non-DIA Control n = 9, DIA Control n = 7, non-DIA *Hif1aCKO* n = 10, DIA *Hif1aCKO* n = 6). Two-Way ANOVA followed by Tukey’s comparison multiple tests were used. Data are mean ± SEM; *P < 0.05, **P < 0.01, ***P < 0.001, ****P < 0.0001; ns = non-significant

To investigate the impact of the combination of the diabetic environment and the *Hif1a-*deficient sympathetic nervous system on the adult heart, we induced diabetes by low doses of STZ treatment, which reduces beta cell mass in the pancreas, and utilized a conditional *Hif1a* deletion mediated by *Isl1*^*Cre*^ (*Hif1aCKO*), which results in altered cardiac sympathetic innervation and formation of sympathetic ganglia [42] (Schematics of experimental study design in Fig. 1A). We focused on the early phase of diabetes-exposure to uncover the effects of the *Hif1a*-deficient sympathetic system on functional and structural adaptations of the diabetic heart. Due to sex differences in cardiac electrophysiological properties [58] and cardiac expressional profiles [17], in this study, we used male mice. After 2 months of the induction of diabetes, blood glucose levels increased in both diabetic groups, with no significant differences observed in relation to *Hif1a* mutation (Fig. 1B). As we previously published, *Hif1aCKO* mice have major malformations of the distal and proximal hindlimbs, reducing the body weight [42]. Accordingly, the body weight of *Hif1aCKO* males was significantly lower compared to control mice (Fig. 1C). The body weight of diabetic *Hif1aCKO* mice was significantly increased compared to non-diabetic *Hif1aCKO* mice. The ratio of heart weight to length of the cranial base (HW/LCB) revealed no significant changes in relative cardiac size (Fig. 1D), which corresponds to an early phase of diabetes exposure. Similarly, observed a moderate decrease in heart rate of diabetic animals were not statistically significant (Fig. 1E). The echocardiographic evaluation of the functional parameters of the left ventricle (LV) revealed a significant reduction in cardiac index (CI), ejection fraction (EF), and fractional shortening (FS) in *Hif1aCKO* compared to non-diabetic control mice, while diabetic control mice did not show any impairment (Fig. 1F–H Additional file 1: Fig. S2). The FS and EF were even more compromised in diabetic *Hif1aCKO* when compared to non-diabetic *Hif1aCKO* mice. These data indicate a negative combinatorial effect of the *Hif1a-*deficient sympathetic nervous system and the diabetic environment. Notably, cardiac performance of diabetic control mice was still preserved at this stage.

### *Hif1a* deletion affects molecular characteristics of sympathetic neurons

**Fig. 2:**
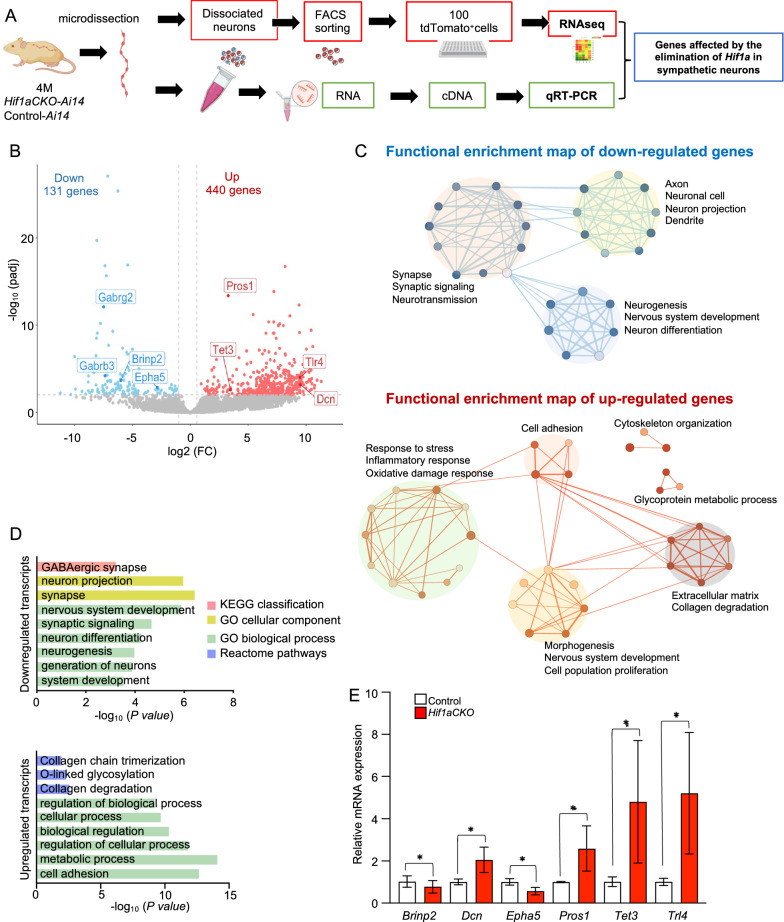
*Hif1a*-mediated transcription signature in sympathetic neurons. **A** Workflow depicts microdissection, dissociation, FACS sorting of single tdTomato^+^ sympathetic neurons for a bulk of 100 cells-RNAseq analysis, and qRT-PCR validation from RNA isolated from the microdissected sympathetic chain from 4-month-old *Hif1aCKO-Ai14* (n = 5) and control-*Ai14* mice (n = 3). **B** The volcano plot shows the change in protein-coding gene expression levels in the *Hif1aCKO-Ai14* compared to control-*Ai14* sympathetic neurons (adjusted p-value < 0.01, and fold change > 1.5 cutoff values). The complete list of identified down- and up-differentially expressed genes is in Additional file 10: Dataset S1. **C** Enrichment map of down- and upregulated gene ontology (GO) sets visualized by the network. Each node represents a GO term; edges depict shared genes between nodes. Each GO set cluster was assigned with representative keywords; a list of GO sets is available in Additional file 10: Dataset S2. **D** The most enriched functional categories for down- and up-regulated genes identified by RNAseq. **E** Validation of relative mRNA expression levels of selected genes by qRT-PCR. mRNA was isolated from the microdissected secondary sympathetic chain of control-*Ai14* (n = 3) and *Hif1aCKO-Ai14* mice (n = 4). Statistical significance assessed by unpaired *t* test: genotype effect (*P < 0.05). *Brinp2*, BMP/retinoic acid inducible neural specific 2; *Dcn*, decorin; *Epha5*, Eph receptor A5; *Pros1*, protein S (alpha); *Tet3*, tet methylcytosine dioxygenase 3; *Tlr4*, toll-like receptor 4

To gain insight at the molecular level on how the *Hif1a* deletion affected molecular characteristics of sympathetic neurons, we sought to identify changes through global transcriptome analysis. We opted to use Bulk-RNA sequencing to obtain sequencing depth and high-quality data [59]. Each biological replicate contained a total of 100 tdTomato^+^ neurons isolated from the adult sympathetic chain ganglia (experimental design in Fig. 2A). Compared to controls, 571 protein-coding genes were differentially expressed in *Hif1aCKO* neurons (Fig. 2B, Additional file 10: Dataset S1). Functional enrichment analysis for downregulated genes revealed highly enriched GO terms associated with nervous system, neuronal function, and neuronal structure, including “synapse”, “synaptic signaling”, “neuron projection”, “neuron differentiation”, and “nervous system development” (Fig. 2C and D, Additional file 10: Dataset S2). The most enriched and specific GO categories for downregulated genes were associated with neurotransmission-related machineries, such as release of neurotransmitters, regulation of synaptic transmission, neuromodulators, and calcium ion binding and signaling, indicating changes in synaptic circuits and neuronal activity. Interestingly, genes encoding components of the GABAergic system, the main inhibitory neurotransmitter system in brain circuits, were downregulated, for example, subunits of the GABA receptor (*Gabrg2* and *Gabrb3*), and modulators of GABA synaptic transmission, *Plcl1* [60], and *Hap1* [61]. In contrast, the most enriched GO category for upregulated genes was the biological process of "cell adhesion". Upregulated genes in this GO category included members of axon guidance molecules and their cognate receptors, semaphorins-plexin (*Sema4g*, *Plxna3*), netrins (*Ntn1*) [62]*;* several members of the cadherin superfamily (*Cdh4, Pcdh11, Pcdhb16, Pcdhb4, Pcdhb14*, *Ctnnal1*) [63]; adhesion-related genes (*Itgae, Itgax, Adgrl4, Sdk2*); and the ephrin receptor (*Ephb4*). The other top enriched GO-terms for upregulated genes in *Hif1aCKO* neurons were related to general cell processes, such as "metabolic process", "regulation of cellular process", and "biological regulation". Interestingly, genes encoding components previously associated with the epidermal growth factor (EGF) receptor-mediated signaling pathway were enriched in *Hif1aCKO* neurons, including *Megf10*, *Sned1*, *Egflam*, *Egfl6*, and *Garem1*. Functional enrichment analyses also identified the upregulation of the Reactome pathway associated with O-linked glycosylation in *Hif1aCKO* sympathetic neurons.

Consistent with the *Hif1aCKO* phenotype, the RNAseq analysis identified differentially expressed genes previously described to be associated with sympathetic neurons, heart function, and hypoxia. For instance, *Prkar2b* is the HIF-1α target gene [64] that influences synaptic function in neurons [65]; *Adra2a* regulates neurotransmitter release from sympathetic nerves and its elimination results in worsened cardiac function [66]; *Brinp1* and *Brinp2* are expressed in sympathetic neurons [67], and *Brinp2* levels are altered after myocardial infarction [68]; and a member of the ephrin receptors, *Epha5,* is important for axon guidance, survival and neurite outgrowth of sympathetic neurons [69] were among downregulated transcripts. Several upregulated genes encoded molecules for axon guidance and axon growth (*Sema4g* [70] and *Dcn* [71]), for sympathetic innervation (*Ntn* [72]), and important for neuroplasticity and heart function (*Tlr4* [73]). Interestingly, activation of TLR4 worsens ischemic injury to the heart and brain [73]. Similarly, *Pros1* is a pro-neurogenic factor and neuroprotectant during ischemic-hypoxic brain injury [74, 75]. We further validated selected genes by qRT-PCR of RNA isolated from the microdissected secondary sympathetic chain (Fig. 2E). Thus, these molecular differences dovetail well with abnormalities in the innervation pattern and worsened heart function of *Hif1aCKO*.

### *Hif1a* deficiency and diabetes affect the sympathetic nervous system

**Fig. 3:**
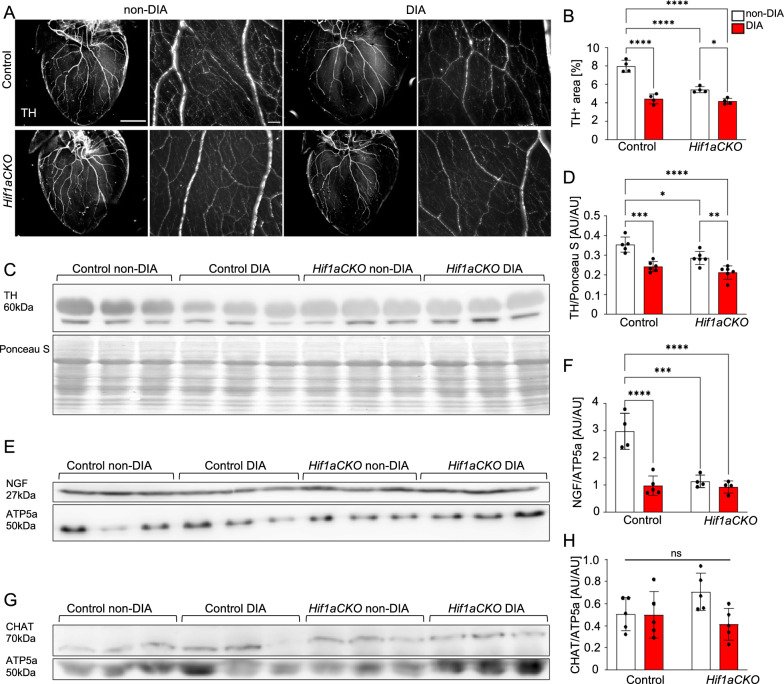
Reduced sympathetic innervation in the diabetic *Hif1aCKO* heart. **A** Representative images of immunohistochemical staining of tyrosine hydroxylase (TH) in the heart (Scale bar = 2 mm), and TH^+^ sympathetic innervation in the heart apex in detail (Scale bar = 100 µm). **B** Positive TH area was quantified using ImageJ and expressed as a percentage of the total heart area. Data are mean ± SD (n = 4). Representative Western blots and quantification of TH (**C**, **D**), nerve growth factor (NGF, **E**, **F**), and choline acetyltransferase (CHAT, **G**, **H**) in the heart. ATP5a or Ponceau S were used as a loading control. Data are mean ± SD (n = 5–6). Statistical significance assessed by two-way ANOVA followed by post hoc Tukey’s multiple comparisons test, *P = 0.0130, **P = 0.0091, ***P = 0.0001, ****P < 0.0001. AU, arbitrary units

Given the notable alterations in the molecular characteristics of sympathetic neurons observed in *Hif1aCKO*, as well as the significant role of sympathetic denervation in cardiac autonomic neuropathy associated with diabetes [10], we proceeded to evaluate the extent of sympathetic innervation in our model. Immunohistochemical staining of tyrosine hydroxylase (TH), a marker of sympathetic neurons, revealed significant reductions in the branching, thickness, and density of sympathetic axons in non-diabetic *Hif1aCKO* hearts when compared to the control group (Fig. 3A, B). The reduced sympathetic innervation was also associated with a decrease in the expression of TH and nerve growth factor (NGF), a vital neurotrophic factor for sympathetic neurons (Fig. 3C–F). Interestingly, the diabetic *Hif1aCKO* mice showed similar levels of sympathetic cardiac denervation, TH and NGF expression as the diabetic control mice, despite the significant impairment of cardiac sympathetic innervation in non-diabetic *Hif1aCKO* mice. The expression of choline acetyltransferase (CHAT), a marker of parasympathetic cholinergic neurons, remained unaffected in the heart (Fig. 3G, H).

**Fig. 4:**
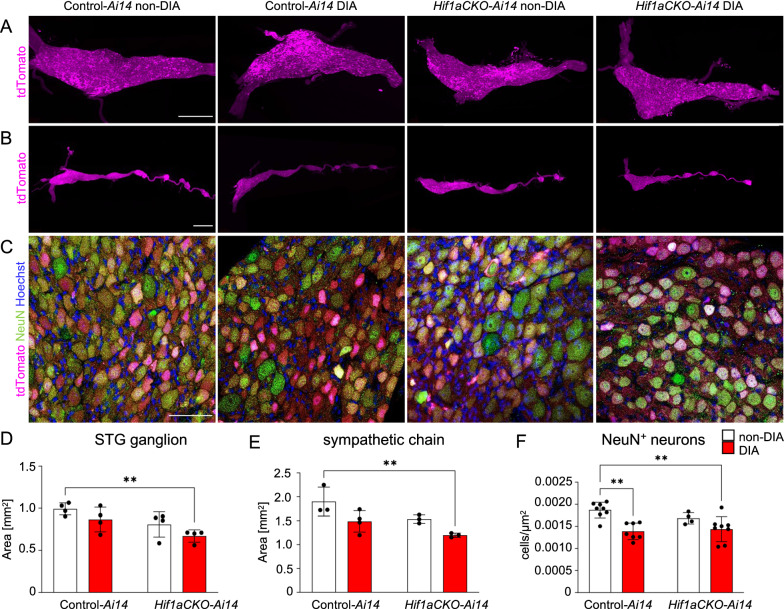
Diminished size of sympathetic chain ganglia by combination of diabetes and *Hif1a* deletion. **A** Representative images of microdissected stellate ganglia (STG) of the secondary sympathetic chain of reporter control-*Ai14* and *Hif1aCKO*-*Ai14* mice. Samples were cleared (CUBIC protocol), imaged, and reconstructed using 3D light-sheet fluorescence microscopy showing *tdTomato*^+^ neurons (see Additional file 2: Video S1, Additional file 3: Video S2, Additional file 4: Video S3, Additional file 5: Video S4). Scale bar 500 µm. **B** Representative images of the secondary sympathetic chain (the STG and four upper ganglia of the thoracic sympathetic chain) reconstructed using 3D light-sheet fluorescence microscopy (see Additional file 6: Video S5, Additional file 7: Video S6, Additional file 8: Video S7, Additional file 9: Video S8). Scale bar 1000 µm. (**C**) Confocal images of immunostaining for NeuN (a marker for differentiated neurons) of *tdTomato*^+^ reporter labeled neurons show neuronal density in transversal section of STG ganglion. Hoechst-stained cell nuclei. Scale bar 50 µm. **D** Quantification of the area of the STG and **E** the area of the sympathetic chain from STG to 4th thoracic ganglion. Data are mean ± SD (n = 3–4). **F** Density of NeuN^+^ cells was quantified per area of the STG (n = 4 samples per genotype, 2 section per sample). Data are mean ± SD. Statistical significance assessed by two-way ANOVA followed by post hoc Tukey ‘s multiple comparisons test **P < 0.01

We next investigated the size of the stellate ganglia, where the majority of cardiac sympathetic postganglionic nerves originate [5]. Using light-sheet fluorescence microscopy and *tdTomato* reporter expression, we generated 3D visualizations of the stellate ganglia and sympathetic chain (Fig. 4A, B). Additional movie files show this in more detail (see Additional file 2: Video S1–Additional file 9: Video S8). The size of the stellate ganglia of diabetic *Hif1aCKO-Ai14* mice was significantly reduced compared to the control-*Ai14*, indicating negative combinatorial effects of *Hif1a* deficiency and diabetes (Fig. 4D). Correspondingly, the size of the sympathetic chain was reduced in diabetic *Hif1aCKO* mice (Fig. 4E). The evaluation of neuronal density in the cross-sectional area of the stellate ganglia demonstrated a decrease in the number of tdTomato^+^/NeuN^+^ neurons in both diabetic control and mutant mice, demonstrating an adverse effect of the diabetic environment (Fig. 4C, F). Overall, the combination of *Hif1a* deficiency and the diabetic environment had an adverse impact on the size of the sympathetic chain ganglia, including the stellate ganglia, which increases the risk of autonomic dysfunction.

**Fig. 5:**
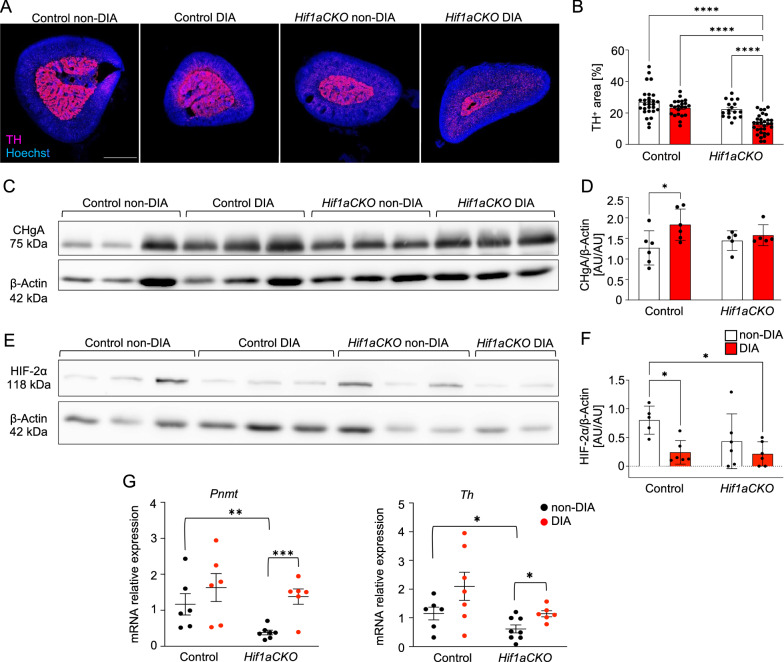
Decreased size of adrenal medulla and altered expression in diabetic *Hif1aCKO* mice. **A** Representative confocal images of cross-sections through the right adrenal glands of control and *Hif1aCKO*, diabetic and non-diabetic adults were labeled with anti-tyrosine hydroxylase (TH). Hoechst-stained cell nuclei. Scale bar = 500 µm. **B** TH^+^ area was quantified using ImageJ and expressed as a percentage of the total adrenal gland area. Data are mean ± SEM (n = 5 gland per genotype and all sections per adrenal gland were used). **C-F** Representative Western blots and quantification of CHgA and HIF-2α protein from the adrenal glands. Beta actin was used as a loading control. Data are mean ± SD (n = 5–6 individual adrenal glands). **G** Relative mRNA expression levels of phenylethanolamine N-methyltransferase (*Pnmt*) and *Th* by qRT-PCR. mRNA was extracted from whole adrenal glands (n = 6–8). Data are mean ± SEM. Statistical significance assessed by two-way ANOVA followed by post hoc Tukey ‘s multiple comparisons test, and unpaired *t*-tests (**G**); *P < 0.05, **P < 0.01, ***P < 0.001, ****P < 0.0001. AU, arbitrary units

Neuroendocrine chromaffin cells of the adrenal medulla are an important component of the sympathoadrenal system in the modulation of metabolic stress responses [76]. Chromaffin cells are modified postganglionic sympathetic neurons that secrete catecholamines directly into the bloodstream. Similar to sympathetic neurons, adrenal chromaffin cells express the catecholamine biosynthetic enzyme, TH. TH positive area of chromaffin cells in the adrenal gland was notably diminished in diabetic *Hif1aCKO* mice compared to all other groups (Fig. 5A, B). We next analyzed expression of chromogranin A, a secretory protein accumulated in chromaffin cell vesicles together with catecholamines. The protein levels of chromogranin A in the adrenal glands were significantly elevated in diabetic control mice but not in diabetic *Hif1aCKO* (Fig. 5C, D). Increased chromogranin A expression has also been reported in patients with type 1 diabetes [77] and it correlates with increased plasma catecholamine levels during the early stages of diabetes [78, 79]. As HIF-2α regulates expression of catecholamine biosynthetic enzymes in adrenal chromaffin cells [80–82], we analyzed the expression of HIF-2α in adrenal glands. The levels of HIF-2α protein were highly variable in *Hif1aCKO,* with undetectable expression in two samples from diabetic and one sample from non-diabetic *Hif1aCKO* (Fig. 5E, F). The diabetic environment was associated with a significant reduction in HIF-2α levels compared to control adrenal glands. Catecholamine biosynthesis in the adrenal medulla depends on the enzyme phenylethanolamine N-methyltransferase (PNMT) that converts norepinephrine to epinephrine and tyrosine hydroxylase (TH) that metabolizes tyrosine. The expression of both the *Pnmt* and *Th* transcripts in chromaffin cells is controlled by HIF-1α and by HIF-2α, although it appears that *Pnmt* is predominantly responsive to HIF-1α, whereas *Th* is responsive to both HIFs in hypoxia [81–83]. Our qRT-PCR analysis performed on mRNA extracted from whole adrenal glands showed the reduced expression of *Pnmt* and *Th* in *Hif1aCKO* compared to control mice (Fig. 5G). Levels of both catecholamine biosynthetic enzymes were increased in diabetic *Hif1aCKO* compared to non-diabetic mutant mice. These results indicate that *Hif1a* deficiency in the sympathoadrenal lineage impacts the size of the adrenal medulla and the secretory capacity of chromaffin cells. This disruption may contribute to the dysregulation of the sympathetic system observed in *Hif1aCKO*. In addition, the combination of diabetes and *Hif1a* deficiency led to a significant reduction in the size of the adrenal medulla, which could potentially alter the production of catecholamines in long-term diabetics.

### Decreased plasma epinephrine levels are related to the *Hif1a*-deficient sympathetic system

**Fig. 6:**
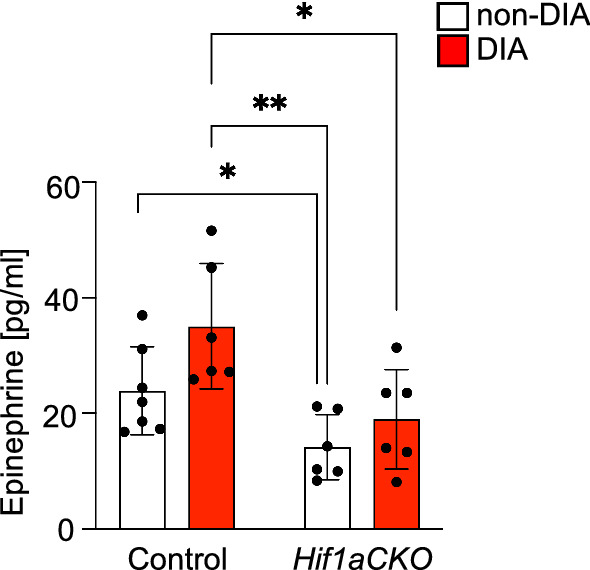
Plasma levels of epinephrine affected by diabetes and by *Hif1a* deficiency. Epinephrine levels in plasma from non-diabetic and diabetic, control and *Hif1aCKO* mice were measured by ELISA (n = 6–7 individual samples per group). Data are mean ± SD. Statistical significance assessed by two-way ANOVA followed by unpaired *t*-test, *P < 0.05, **P < 0.01

Adrenal chromaffin cells predominantly produce epinephrine [84]. To characterize the impact of diabetes and *Hif1a* deficiency, we analyzed potential changes in secretion of epinephrine by measuring epinephrine levels in plasma of non-diabetic and diabetic, control and *Hif1aCKO* mice. Along with reduced *Pnmt* mRNA in the adrenal glands, *Hif1aCKO* mice exhibited significantly lower plasma levels of epinephrine than control mice (Fig. 6). Compared plasma epinephrine levels in diabetic controls, diabetic *Hif1aCKO* mice showed significantly lower epinephrine levels, suggesting that a loss of *Hif1a* in the sympathoadrenal cell lineage altered epinephrine production in response to the diabetic environment.

### *Hif1aCKO* mice display cardiac remodeling of increased AGE accumulation and profibrotic response

**Fig. 7:**
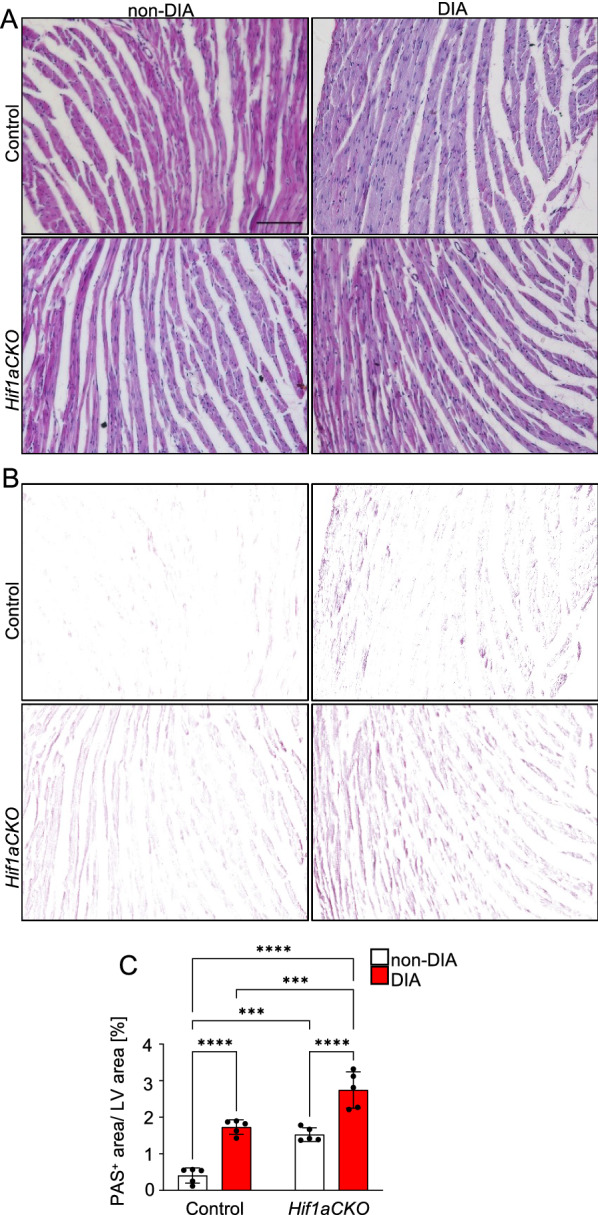
Increased advanced glycation end products in the left ventricle of diabetic *Hif1aCKO* hearts. **A** Representative images of PAS staining of advanced glycation end products (AGEs). Scale bar 50 µm. **B** Delineated PAS^+^ area in the LV myocardium by Adobe Photoshop. **C** PAS^+^ area was quantified using ImageJ and expressed as a percentage of the area of the LV. Data are mean ± SD (n = 5 hearts per genotype and one section per sample). Statistical significance assessed by two-way ANOVA followed by post hoc Tukey ‘s multiple comparisons test, ***P < 0. 001, ****P < 0.0001. LV, left ventricle

We next investigated whether the *Hif1a*-deficient sympathetic nervous system affected the progression of diabetic cardiomyopathy. Diabetic cardiomyopathy is initially characterized by LV functional diastolic abnormalities and structural remodeling, including myocardial fibrosis, inflammation, and coronary microvascular dysfunction [9, 85]. Hyperglycemia and increased levels of glucose metabolites upregulate the production of advanced glycation end products (AGEs), affecting the structural and functional characteristics of cardiomyocytes and endothelial cells [86]. The sympathetic nervous system increases glucose uptake in peripheral tissues, and thus, dysfunction of the sympathetic system impairs glucose metabolism and uptake in target organs (as reviewed in [87–89]). Although AGE formation is primarily driven by hyperglycemia, oxidative stress can exacerbate the process [90, 91]. To explore the potential relationships between the *Hif1aCKO* dysfunctional sympathetic system and AGE formation in the heart, we assessed AGE accumulation by Periodic acid-Schiff staining. Interestingly, we found significantly higher AGE accumulation in the LV of non-diabetic *Hif1aCKO* compared to non-diabetic controls (Fig. 7). As expected, our analysis demonstrated a significant increase in AGE accumulation in both diabetic control and *Hif1aCKO* compared to the non-diabetic control heart. Moreover, diabetic *Hif1aCKO* mice exhibited the most abundant AGE accumulation in the LV. These results suggest that the dysfunctional sympathetic system contributes to enhanced AGE production, which may accelerate the development of diabetic cardiomyopathy and compromise cardiac function in *Hif1aCKO* mice.

**Fig. 8:**
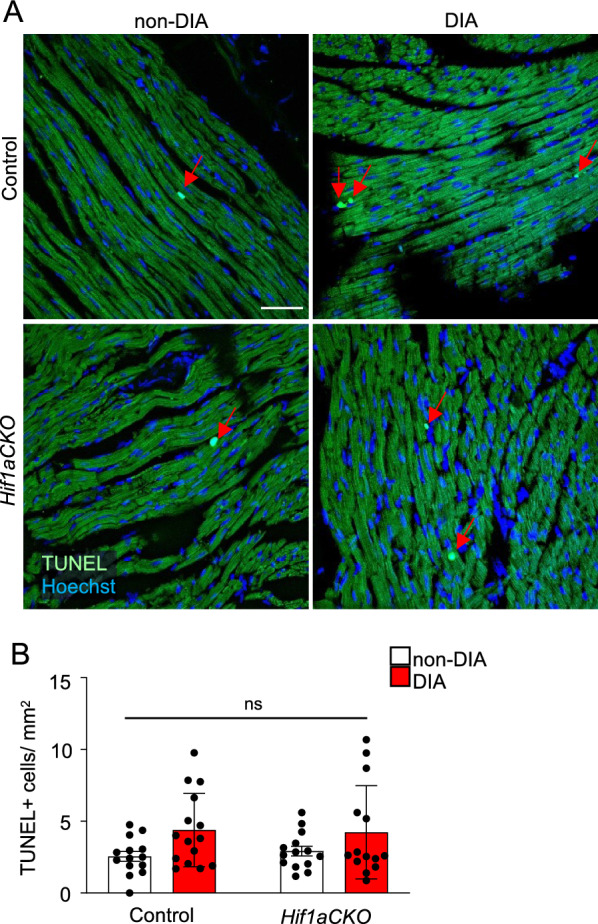
Increased apoptotic cells in the diabetic hearts. **A** Representative images of TUNEL staining with Hoechst-stained cell nuclei. Red arrows indicate TUNEL^+^ cells. Scale bar 50 µm. **B** Quantification of TUNEL^+^ apoptotic cells per mm^2^ of the LV. Data are mean ± SEM (n = 5 individuals per genotype and 3 sections per group). Statistical significance assessed by two-way ANOVA (diabetes effect F_1,53_ = 6.891, P = 0.0113) followed by post hoc Tukey’s multiple comparisons test (*P* = not significant, ns). LV, left ventricle

Systemic hyperglycemia and impaired LV function are associated with increased cell death and profibrotic responses. No significant differences in the number of apoptotic cells were found in the LV among the experimental groups (Fig. 8). These results are consistent with an early phase of diabetes-exposure model, as the extent of cardiomyocyte death parallels the severity and stage of diabetes.

**Fig. 9:**
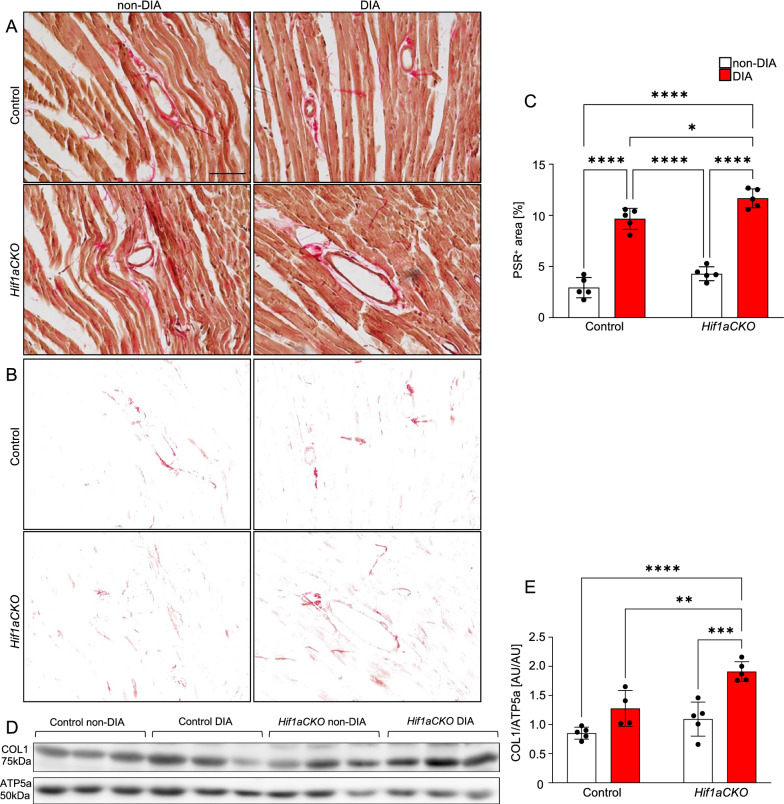
Collagen levels in the heart affected by the combination of diabetes and *Hif1a* mutation. **A** Representative images of picrosirius red staining (PSR) of collagen in the LV and **B** delineated collagen^+^ area in the LV myocardium using Adobe Photoshop. Scale bar = 50 µm. **C** PSR^+^ area was quantified using ImageJ and expressed as a percentage of the area of the LV. Data are mean ± SD (n = 5 hearts per genotype and one section per sample). **D**, **E** Representative Western blots and quantification of collagen type I (COL1) in the whole heart. Data are mean ± SD (n = 5). Statistical significance assessed by two-way ANOVA followed post hoc Tukey ‘s multiple comparisons test, *P < 0.05, **P < 0.01, ***P < 0.001, ****P < 0.0001. LV, left ventricle; AU, arbitrary units

Myocardial fibrosis in diabetic cardiomyopathy involves accumulation of type I and III collagen and its crosslinking, perivascular and interstitial fibrosis, and thickened small coronary vessels [9, 92]. We found significantly increased cardiac collagen deposition in the LV area of diabetic animals (Fig. 9A−C). Furthermore, diabetic *Hif1aCKO* animals showed significantly larger collagen accumulation compared to diabetic control hearts, indicating a synergistic effect of *Hif1a-*deficient sympathetic system and diabetes on collagen deposition. In addition, we quantified the protein levels of collagen type I, the major component of the myocardial extracellular matrix [93]. When compared to the other groups, the hearts of diabetic *Hif1aCKO* mice showed the highest levels of collagen type 1 (Fig. 9D, E). These findings suggest an escalation of myocardial fibrosis, and consequently, the development of pathological changes associated with diabetic cardiomyopathy.

### Combination of diabetes and the *Hif1a*-deficient sympathetic system exacerbates myocardial damage

**Fig. 10:**
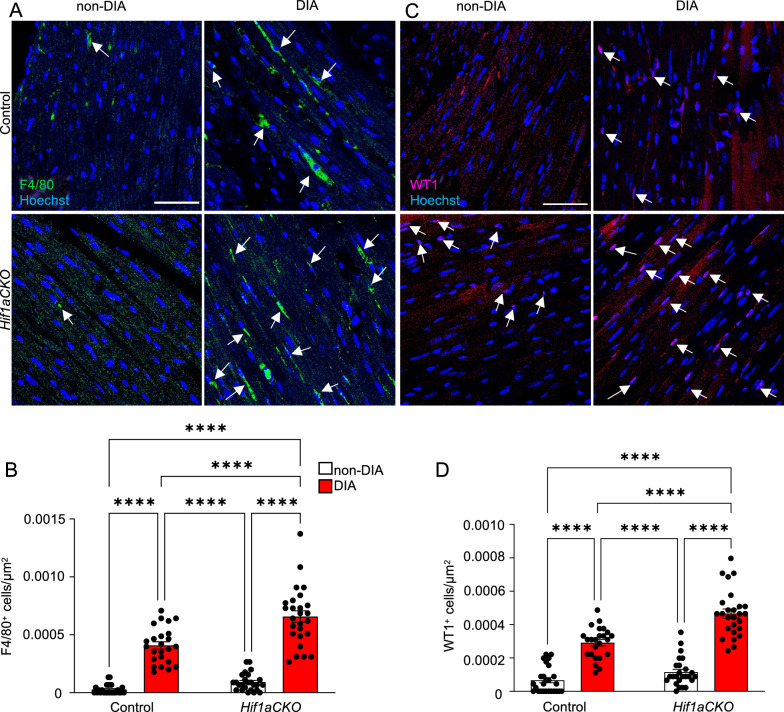
Adverse tissue remodeling in the myocardium of the left ventricle of diabetic *Hif1aCKO*. **A** Representative confocal images of immunohistochemical staining of macrophages (F4/80) and **C** WT1 with Hoechst-stained cell nuclei in the LV of the heart. White arrows indicate F4/80^+^ cells and WT1^+^ cells. Scale bars 50 µm. **B** Quantification of number of F4/80^+^ cells and **D** WT1^+^ cells in the LV. Data are mean ± SEM (n = 4 samples per genotype, one section per sample and 6 areas per section). Statistical significance assessed by two-way ANOVA followed by post hoc Tukey ‘s multiple comparisons test ****P < 0.0001. LV, left ventricle

Profibrotic responses and increased production of AGEs trigger cardiomyocyte and endothelial cell damage, and infiltration of inflammatory cells into the myocardium have been linked to cardiac injury [8]. As macrophages are essential cells in myocardial injury repair and remodeling [94, 95], we evaluated the presence of these cells in the LV myocardium of our model. Our results showed the largest macrophage infiltration in the diabetic *Hif1aCKO* heart (Fig. 10A, B). Although both diabetic control and mutant hearts had significantly more F4/80^+^ infiltrating macrophages than non-diabetic hearts, the combination of diabetes and the *Hif1a-*deficient sympathetic system resulted in the most extensive macrophage recruitment. In addition, the number of cells expressing the WT1 transcription factor in the LV myocardium was significantly increased in diabetic hearts (Fig. 10C, D). Importantly, the diabetic *Hif1aCKO* heart had a significantly higher number of WT1 positive cells compared to the diabetic control heart. Upregulation of WT1 expression in the adult myocardium has been reported to promote vascularization and cardiac remodeling following myocardial infarction [96, 97]. Taken together, our findings suggest that a dysfunctional sympathetic nervous system can exacerbate cardiac injury and adverse remodeling in the diabetic heart.

## Discussion

In this study, we uncovered a previously undescribed protective role of HIF-1α in the sympathetic system over the progression of diabetic cardiomyopathy in genetically modified *Hif1aCKO* mice. We employed an early phase of diabetes-exposure model to investigate the effects of the *Hif1a*-deficient sympathetic system on functional and structural adaptations of the diabetic heart. Longitudinal exposure of *Hif1aCKO* mice to diabetes, spanning 2 months, revealed that the combination of diabetes and *Hif1a* deficiency in the sympathetic nervous system resulted in compromised cardiac performance and accelerated adverse myocardial remodeling.

Hallmarks of diabetic cardiomyopathy are structural remodeling and compromised systolic or diastolic function in the absence of other cardiac risk factors, representing high risk of sudden cardiac death [7–9, 98]. The diabetic environment of hyperglycemia and metabolic dysregulation induces adverse cardiac remodeling characterized by increased fibrosis, vascular dysfunction, inflammation, increased AGEs, and cardiomyocyte death. These pathophysiological changes, and the progression of diabetic heart disease and heart failure have been associated with the impairment of the sympathetic nervous system [12–14]. Initially, the sympathetic nervous system is hyperactive with elevated systemic and regional norepinephrine spillover, but eventually with the progression of diabetes, sustained sympathetic activation depresses the sympathetic nervous system sensitivity, and results in sympathetic denervation in the diabetic heart [10, 12, 13, 99]. Advanced cardiac sympathetic denervation correlates with high mortality rates in diabetic patients [13, 100]. Although the etiology of cardiac denervation in diabetes is not fully understood, several mechanisms have been implicated, such as abnormalities in the neurotropic factors, prolonged exposure to catecholamines, microvascular complications, formation of AGEs, and nerve hypoxia [14, 101–104].

A key role of HIF-1α in the development of sympathetic neurons and sympathetic innervation of the heart has only recently been demonstrated [42]. HIF-1α is also required for sympathetic nervous system activation mediated by the carotid body [37]. Although diabetes is associated with increased tissue hypoxia, optimal activation of HIF-1α is blunted in the diabetic environment, affecting the adaptive hypoxic responses ([21–25], reviewed in [26, 27]). Given the far-reaching effects of the HIF-1 transcription factor regulating hundreds of genes, the impaired activation of HIF-1α would alter cellular and systemic adaptive responses to diabetic milieu, including vasomotor regulation, apoptosis, angiogenesis, erythropoiesis, cell growth, and metabolism [15, 16]. Understanding the nature of modulation of diabetic complications by HIF-1α is highly relevant for the clinical translatability. For example, the detrimental roles of reduced activation of HIF-1α contributes to the development of diabetic wounds [21], adverse post-ischemic myocardial remodeling [24], the acceleration of diabetic cardiomyopathy [24, 28], and renal injury [55]. Only recently, it was reported that a loss of *Hif1a* in sensory neurons leads to a faster progression of peripheral neuropathy in diabetic mice [103]. However, the role of HIF-1α in the impairment of the sympathetic nervous system caused by metabolic disturbances of diabetic milieu remains unclear.

We established for the first time that the elimination of *Hif1a* altered molecular characteristics of sympathetic neurons of adult mice, including the downregulation of genes/pathways associated with the structure of synapses and synaptic signaling, neuronal projections, and neurotransmission machinery. These changes indicate that HIF-1α is important for the maintenance of functional features of sympathetic neurons. We have previously shown that *Hif1a* deficiency in sympathetic neurons results in reduced sympathetic innervation in the heart and the functional impairment of the left ventricle parameters [42]. Therefore, we hypothesized that the *Hif1a*-deficient sympathetic nervous system would accelerate diabetic cardiomyopathy. The compromised heart performance, demonstrated by reduced fractional shortening and ejection fraction, in diabetic *Hif1aCKO* mice compared to other groups further support this notion. We then investigated the effects of the diabetic environment on the sympathetic system. The sympathoadrenal system participates in both circulatory and metabolic control, and diabetes represents conditions with different degrees of metabolic derangement. Interestingly, we found that although cardiac sympathetic innervation was significantly impaired in the *Hif1aCKO* heart, there was a similar level of sympathetic denervation in both diabetic *Hif1aCKO* and diabetic control hearts. These results are somewhat surprising and require further investigation. One possible explanation is that the sympathetic denervation in the *Hif1aCKO* heart had already reached the limit for survival, and additional denervation as a result of the diabetic influence was prevented by unknown mechanisms. Furthermore, our study confirms previous findings [79] that significant sympathetic denervation is present in the diabetic heart during the early stages of diabetes, even in the absence of detectable LV dysfunction in diabetic controls. These findings suggest that sympathetic denervation may be an early marker of diabetic heart disease and may contribute to the development of cardiac dysfunction over time. However, we found that the combination of *Hif1a* deficiency and the diabetic environment had an adverse impact on the size of the sympathetic chain ganglia, including the stellate ganglia, where most of the sympathetic nerves projecting to the heart originate. In addition, *Hif1a* deficiency in the sympathoadrenal lineage resulted in reduced adrenal medulla size and altered secretory capacity of chromaffin cells in *Hif1aCKO* diabetic mice. Consequently, the combination of diabetes and *Hif1a*-deficient sympathetic system exacerbates adverse cardiac remodeling, demonstrated by the escalated accumulation of AGEs and collagens, macrophage infiltration, and increased expression of cardiac injury markers.

We found the highest accumulation of AGEs in the LV of diabetic *Hif1aCKO* mice. Given that non-enzymatic glycation reactions alter proteins, lipids, and DNA, the presence of AGEs is consider a diabetogenic risk factor in diabetes-associated complications [9, 104, 105]. Therefore, the increased production of AGEs in *Hif1aCKO* mice may accelerate the development of diabetic cardiomyopathy and compromise cardiac function. Unexpectedly, we found enhanced AGE accumulation in non-diabetic *Hif1aCKO* as compared to the control heart. The formation of AGEs is widely acknowledged to be mainly driven by hyperglycemia. In addition, oxidative stress can exacerbate the production of AGEs [90, 91]. Considering that the sympathetic system plays a crucial role in modulating glucose metabolism of target organs (as reviewed in [87, 88]), it is reasonable to hypothesize that a dysfunctional sympathetic system could potentially have an impact on glucose metabolism, glucose uptake, and/or lead to increased oxidative stress in target tissues, which could in turn exacerbate the production of AGEs. These findings indicate that an impaired sympathetic system may contribute to AGE-mediated pathologies, including neurodegenerative and metabolic diseases [91]. Together with increased AGE deposition, we detected an increased production and accumulation of collagens in the diabetic *Hif1aCKO* heart. Collagen deposition is a hallmark of fibrosis, which is another important process of structural remodeling in diabetic cardiomyopathy [9, 85, 92]. Enhanced fibrosis and increase deposition of AGEs contributes to left ventricular stiffness of the diabetic heart and to the pathogenesis of heart failure [85]. Consistent with enhanced structural adverse remodeling in *Hif1aCKO* diabetic hearts, we found an increased number of infiltrating F4/80^+^ macrophages and upregulated levels of myocardial WT1, indicating an activation of responses to cardiac injury [95–97].

In conclusion, our results indicate that diabetic hearts with the *Hif1a* deficient cardiac sympathetic nervous system are less able to adapt in the longer term to the adverse diabetic environment. We propose that HIF-1α regulation is important for the maintenance of functional features of sympathetic neurons in physiological and pathophysiological conditions. Consequently, HIF-1α dysregulation may contribute to various sympathetic abnormalities underlying cardiac pathologies, including heart failure and sudden cardiac death.

## Supplementary Information

**Additional file 1: Table S1. **Primer sequences for genotyping. **Table S2. **Primer sequences for quantitative real-time polymerase chain reaction. **Table S3. **Primary antibodies. **Figure S1.** Fluorescent Activated Cell Sorting of tdTomato^+^ neuronal cells. Gating strategy of the tdTomato^+^ (A) and tdTomato^-^ sympathetic neurons (B). **Figure S2.** Basal left ventricular echocardiographic parameters. The echocardiographic evaluation of left ventricular systolic function of non-diabetic and diabetic, control and *Hif1aCKO* mice (non-DIA Control n = 9, DIA Control n = 7, non-DIA *Hif1aCKO* n = 10, DIA *Hif1aCKO* n = 6). Two-Way ANOVA followed by Tukey’s comparison multiple tests were used. Data are mean ± SEM; *P < 0.05, **P < 0.01, ***P < 0.001, ****P < 0.0001; ns = non-significant. Abbreviations: AWTd, diastolic anterior wall thickness; AWTs, systolic anterior wall thickness; LV, left ventricle; LVDd, diastolic cavity diameter; LVDs, systolic cavity diameter; PWTd, diastolic posterior wall thickness; PWTs, systolic posterior wall thickness; RWT, relative wall thickness; SV, stroke volume.

**Additional file 2: Video S1.** Non-diabetic control stellate ganglion. Microdissected stellate ganglion of *tdTomato *reporter control-*Ai14* mice was cleared (CUBIC protocol), imaged, and reconstructed in 3D using light-sheet fluorescence microscopy (LFSM). Video shows the distribution tdTomato^+^ sympathetic neurons in the anatomical microenvironment of the ganglion.

**Additional file 3:** **Video S2.** Diabetic control stellate ganglion. Microdissected stellate ganglion of diabetic *tdTomato* reporter control-*Ai14* mice was cleared (CUBIC protocol), imaged, and reconstructed in 3D using light-sheet fluorescence microscopy (LFSM). Video shows the distribution tdTomato^+^ sympathetic neurons in the anatomical microenvironment of the ganglion.

**Additional file 4: Video S3.** Non-diabetic *Hif1aCKO* stellate ganglion. Microdissected stellate ganglion of *tdTomato* reporter *Hif1aCKO-Ai14* mice was cleared (CUBIC protocol), imaged, and reconstructed in 3D using light-sheet fluorescence microscopy (LFSM). Video shows the distribution tdTomato^+^ sympathetic neurons in the anatomical microenvironment of the ganglion.

**Additional file 5:** **Video S4.** Diabetic *Hif1aCKO* stellate ganglion. Microdissected stellate ganglion of diabetic *tdTomato* reporter *Hif1aCKO-Ai14* mice was cleared (CUBIC protocol), imaged, and reconstructed in 3D using light-sheet fluorescence microscopy (LFSM). Video shows the distribution tdTomato^+^ sympathetic neurons in the anatomical microenvironment of the ganglion.

**Additional file 6: Video S5.** The secondary sympathetic chain of non-diabetic control. Microdissected the stellate and four upper ganglia of the thoracic sympathetic chain of *tdTomato* reporter control-*Ai14* mice were cleared (CUBIC protocol), imaged, and reconstructed in 3D using light-sheet fluorescence microscopy (LFSM). Video shows the distribution tdTomato^+^ sympathetic neurons in the anatomical microenvironment of the ganglion.

**Additional file 7: Video S6.** The secondary sympathetic chain of diabetic control. Microdissected the stellate and four upper ganglia of the thoracic sympathetic chain of diabetic *tdTomato* reporter control-*Ai14* mice were cleared (CUBIC protocol), imaged, and reconstructed in 3D using light-sheet fluorescence microscopy (LFSM). Video shows the distribution tdTomato^+^ sympathetic neurons in the anatomical microenvironment of the ganglion.

**Additional file 8:** **Video S7.** The secondary sympathetic chain of non-diabetic *Hif1aCKO*. Microdissected the stellate and four upper ganglia of the thoracic sympathetic chain of tdTomato reporter *Hif1aCKO-Ai14* mice were cleared (CUBIC protocol), imaged, and reconstructed in 3D using light-sheet fluorescence microscopy (LFSM). Video shows the distribution tdTomato^+^ sympathetic neurons in the anatomical microenvironment of the ganglion.

**Additional file 9:** **Video S8.** The secondary sympathetic chain of diabetic *Hif1aCKO*. Microdissected the stellate and four upper ganglia of the thoracic sympathetic chain of diabetic *tdTomato* reporter *Hif1aCKO-Ai14* mice were cleared (CUBIC protocol), imaged, and reconstructed in 3D using light-sheet fluorescence microscopy (LFSM). Video shows the distribution tdTomato^+^ sympathetic neurons in the anatomical microenvironment of the ganglion.

**Additional file 10:** **Dataset S1.** RNA sequencing: differentially expressed protein coding genes (adjusted P-value padj < 0.01, fold change (FC) > 1.5 cutoff values). **Dataset S2.** g: Profiler analyses of differentially expressed genes.

## Data Availability

All data generated or analyzed during this study are included in this published article. The raw RNAseq data for this study were deposited at GEO (https://www.ncbi.nlm.nih.gov/geo/).
