# Supplementary material for: Dysregulation of hypoxia-inducible factor 1α in the sympathetic nervous system accelerates diabetic cardiomyopathy
Source: Cardiovasc Diabetol. 2023 Apr 18;22:88. doi: 10.1186/s12933-023-01824-5 (PMC10114478; doi:10.1186/s12933-023-01824-5)
Supplement: Supplementary file 1 — Additional file 1: Table S1. Primer sequences for genotyping. Table S2. Primer sequences for quantitative real-time polymerase chain reaction. Table S3. Primary antibodies. Figure S1. Fluorescent Activated Cell Sorting of tdTomato+ neuronal cells. Gating strategy of the tdTomato+ (A) and tdTomato- sympathetic neurons (B). Figure S2. Basal left ventricular echocardiographic parameters. The echocardiographic evaluation of left ventricular systolic function of non-diabetic and diabetic, control and Hif1aCKO mice (non-DIA Control n = 9, DIA Control n = 7, non-DIA Hif1aCKO n = 10, DIA Hif1aCKO n = 6). Two-Way ANOVA followed by Tukey’s comparison multiple tests were used. Data are mean ± SEM; *P < 0.05, **P < 0.01, ***P < 0.001, ****P < 0.0001; ns = non-significant. Abbreviations: AWTd, diastolic anterior wall thickness; AWTs, systolic anterior wall thickness; LV, left ventricle; LVDd, diastolic cavity diameter; LVDs, systolic cavity diameter; PWTd, diastolic posterior wall thickness; PWTs, systolic posterior wall thickness; RWT, relative wall thickness; SV, stroke volume. [file 12933_2023_1824_MOESM1_ESM.pdf]

Dysregulation of hypoxia-inducible factor 1a in the sympathetic nervous system accelerates diabetic cardiomyopathy

Hrabalova P, Bohuslavova R, Matejkova K, Papousek F, Sedmera D, Abaffy P, Kolar F, and Pavlinkova G

Additional Information

1. **Additional Tables S1–S4**
2. **Additional Figures S1, S2**
3. **Legends for Video S1–S8**

Table S1: Primer sequences for genotyping

Gene symbol	Forward primer sequence	Reverse primer sequence
Hif1a flox	5'-TGCATGTGTATGGGTGTTTTG-3'	5'-GAAAACTGTCTGTAACTTCATTTCC-3'
Isl1/Cre	5’-GCCTGCATTACCGGTCGATGCAACGA-3'	5’-GTGGCAGATGGCGCGGCAACACCATT-3'
WT-TomatoAi14	5´ AAGGGAGCTGCAGTGGAGTA 3´	5´ CCGAAAATCTGTGGGAAGTC 3´
Transgene-TomatoAi14	5´ CTGTTCCTGTACGGCATGG 3´	5´ GGCATTAAAGCAGCGTATCC 3´

Abbreviations: *Hif1a*, Hypoxia inducible factor 1 alpha; *Isl1*, ISL1 transcription factor, LIM/homeodomain

Table S2: Primer sequences for quantitative real-time polymerase chain reaction

Gene symbol	Forward primer sequence	Reverse primer sequence
Ache	5'-ACCTGTGGGCTCACGTAGATT-3'	5'-CCACGTACTGGTAGCAGACATT-3'
Adra2a	5'-AGCTGCAAGATCAACGACCA-3'	5'-ACGCTTGGCGATCTGGTAAA-3'
Brinp2	5'-GAGTCTCCTCGCAGTCCTTG-3'	5'-CTTCTCCCAGGCGGTTAGTG-3'
Cckar	5'-GCCAGCACTTGGTAGGAAGC-3'	5'-AAGGAATATGAAGGAGTACAGGAGA-3'
DCN	5'-GAGGGCTCCTGTGGCAAAT-3'	5'-ATCAGGGGATTGTCAGGGTC-3'
Gabrg2	5'-AGAAAAACCCTCTTCTTCGGATG-3'	5'-GTGGCATTGTTCATTTGAATGGT-3'
Garem1	5'-CAGGTGCGACACCTTGAGAG-3'	5'-TTCAGGAGCCGGCATTCTTC-3'
Epha5	5'-CTGGCGGACGGAAAGATGT-3'	5'-TTCAGGCCGATTTGCTGGG-3'
Hprt1	5'-GCTTGCTGGTGAAAAGGACCTCTCGAAG-3'	5'-CCCTGAAGTACTCATTATAGTCAAGGGCAT-3'
Pnmt	5'-GGAGACCTGAGCAACCCTGA-3'	5'-TCCTGACGGTTGACTTCCAA-3'
Pros1	5'-CGGCCATCCTCTCAGCAATG-3'	5'-AGGACTTGTGAAGCACGCTC-3'
Sctr	5'-GTGGAAGTGCCATGTCCGAA-3'	5'-GCCTGGGGAAGGTTTCTGAC-3'
Svop	5'-TCTGTTCCAGCTCAGGCAGTT-3'	5'-TGATCAGTTGACAGTTGGACGG-3'
Syt9	5'-CATCTACCACCTGCGGGACC-3'	5'-GACCACAGGCAGTAACCACG-3'
Tet3	5'-AAACTGAGCACGCCAGAGAA-3'	5'-CAGCACCGAGTAGCTTTCCA-3'
Tlr4	5'-CGCTGCCACCAGTTACAGAT-3'	5'-TCTGATCCATGCATTGGTAGGT-3'
Th	5'-CCAGTGAAATTAGGCTCCCTG-3'	5'-CTCTCCTCGAATACCACAGCC-3'

*Primers were designed using Primer3 and BLAST software. Abbreviations: *Ache*, acetylcholinesterase; *Adra2a*, adrenergic receptor, alpha 2a; *Brinp2*, BMP/retinoic acid inducible neural specific 2; *Cckar*, cholecystokinin A receptor; *DCN*, decorin; *Gabrg2*, gamma-aminobutyric acid (GABA) A receptor, subunit gamma 2; *Garem1*, GRB2 associated regulator of MAPK1 subtype 1; *Epha5*, Eph receptor A5; *Hprt1*, hypoxanthine guanine phosphoribosyl transferase; *Pnmt*, Phenylethanolamine N-methyltransferase; *Pros1*, protein S (alpha); *Sctr*, secretin receptor; *Svop*, SV2 related protein; *Syt9*, synaptotagmin IX; *Tet3*, tet methylcytosine dioxygenase 3; *Tlr4*, toll-like receptor 4; *Th*, tyrosine hydroxylase.

Table S3: Primary antibodies

**Primary antibody against**	Host species	**Company**	**Catalog number**	**WB dilution**	**IHC dilution**
beta actin (13E5)	rabbit HRP conjungate	Cell Signaling	5125	1:5000	
CHAT	goat	Merck Millipore	AB144P	1:1000	
CHgA	rabbit	Abcam	ab15160	1:2000	1:100
Col1	rabbit	MD bioproducts	20302	1:1000	
F4/80	rat	Biorad	MCA497R		1:500
HIF-2 alpha /EPAS1 (ep190b)	mouse	Novus biologicals	NB100-132	1:500	
NeuN	rabbit	Abcam	ab177487		1:500
NGF	rabbit	Abcam	ab52918	1:1000	
TH	rabbit	Merck Millipore	ab152	1:1000	1:750
WT1	rabbit	Calbiochem	CA1026		1:200
Membrane Fraction WB Cocktail		Abcam	ab140365	1:200 primary antibody 1:2500 secondary antibody	

Abbreviations: CHAT, choline acetyltransferase; CHgA, chromogranin A; Col1, collagen type I; NeuN, neuronal nucleus marker; NGF, nerve growth factor; TH, tyrosine hydroxylase; VEGFA, vascular endothelial growth factor; WT1, Wilms tumor 1.

Table S4: Secondary antibodies

**Secondary antibody against**	**Host species**	**Company**	***Catalog number***	**Dilution**
Alexa Fluor® **488**-conjugated AffiniPure Donkey Anti-**Mouse** IgG (H+L)	donkey	Jackson ImmunoResearch	715-545-150	1:500
Alexa Fluor® **488**-conjugated AffiniPure Donkey Anti-**Rabbit** IgG (H+L)	donkey	Jackson ImmunoResearch	715-545-152	1:500
Alexa Fluor® **488**-conjugated AffiniPure Fab Fragment Donkey Anti-**Rat** IgG (H+L)	donkey	Jackson ImmunoResearch	712-547-003	1:500
Alexa Fluor® **594**-conjugated AffiniPure Donkey Anti-**Rabbit** IgG (H+L)	donkey	Jackson ImmunoResearch	711-585-152	1:500
Alexa Fluor® **647**-conjugated AffiniPure Donkey Anti-**Mouse** IgG	donkey	Jackson ImmunoResearch	715-605-151	1:500
Anti-**Mouse** IgG-Peroxidase	rabbit	Sigma	A9044	1:10000
Anti-**Rabbit** IgG-Peroxidase	goat	Sigma	A0545	1:10000

A

B

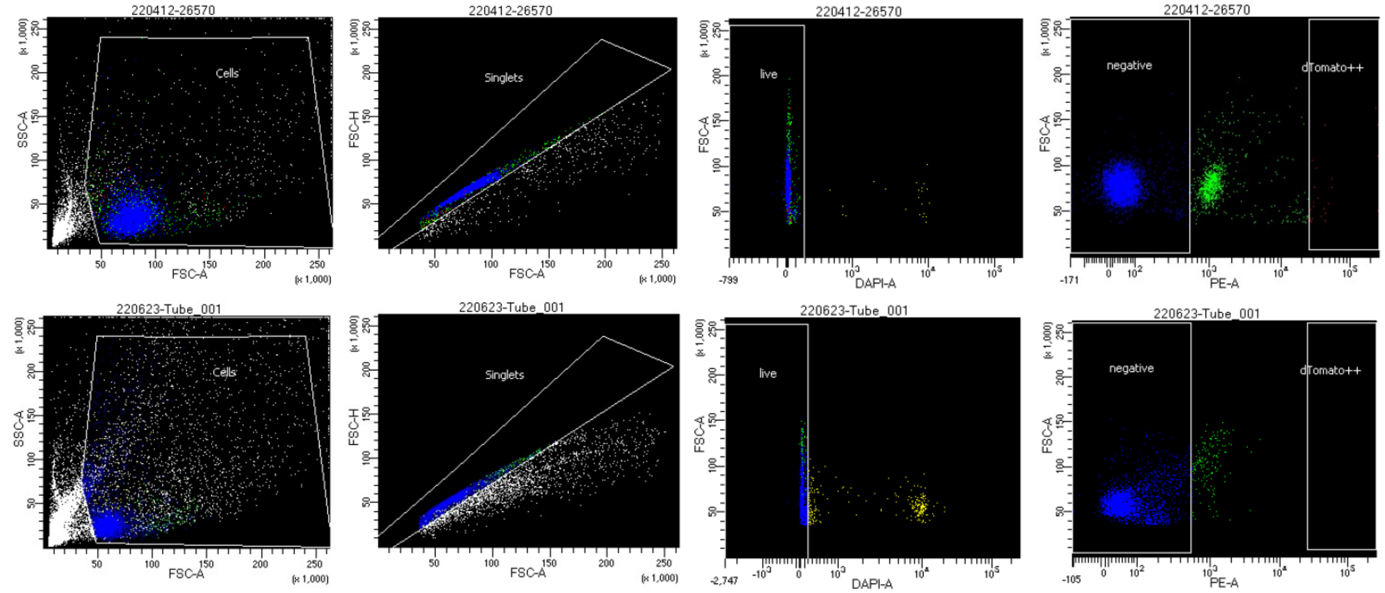

Tube: 26570

Population	#Events	%Parent	%Total
All Events	10,000	####	100.0
Cells	5,310	53.1	53.1
Singlets	4,817	90.7	48.2
live	4,795	99.5	47.9
negative	3,910	81.5	39.1
dTomato++	25	0.5	0.2

Tube: Tube_001

Population	#Events	%Parent	%Total
All Events	20,000	####	100.0
Cells	4,801	24.0	24.0
Singlets	3,135	65.3	15.7
live	2,892	92.2	14.5
negative	2,748	95.0	13.7
dTomato++	0	0.0	0.0

Figure S1. Fluorescent Activated Cell Sorting of tdTomato^{+} neuronal cells
Gating strategy of the tdTomato $^{+}$ (**A**) and tdTomato^{-} sympathetic neurons (**B**).

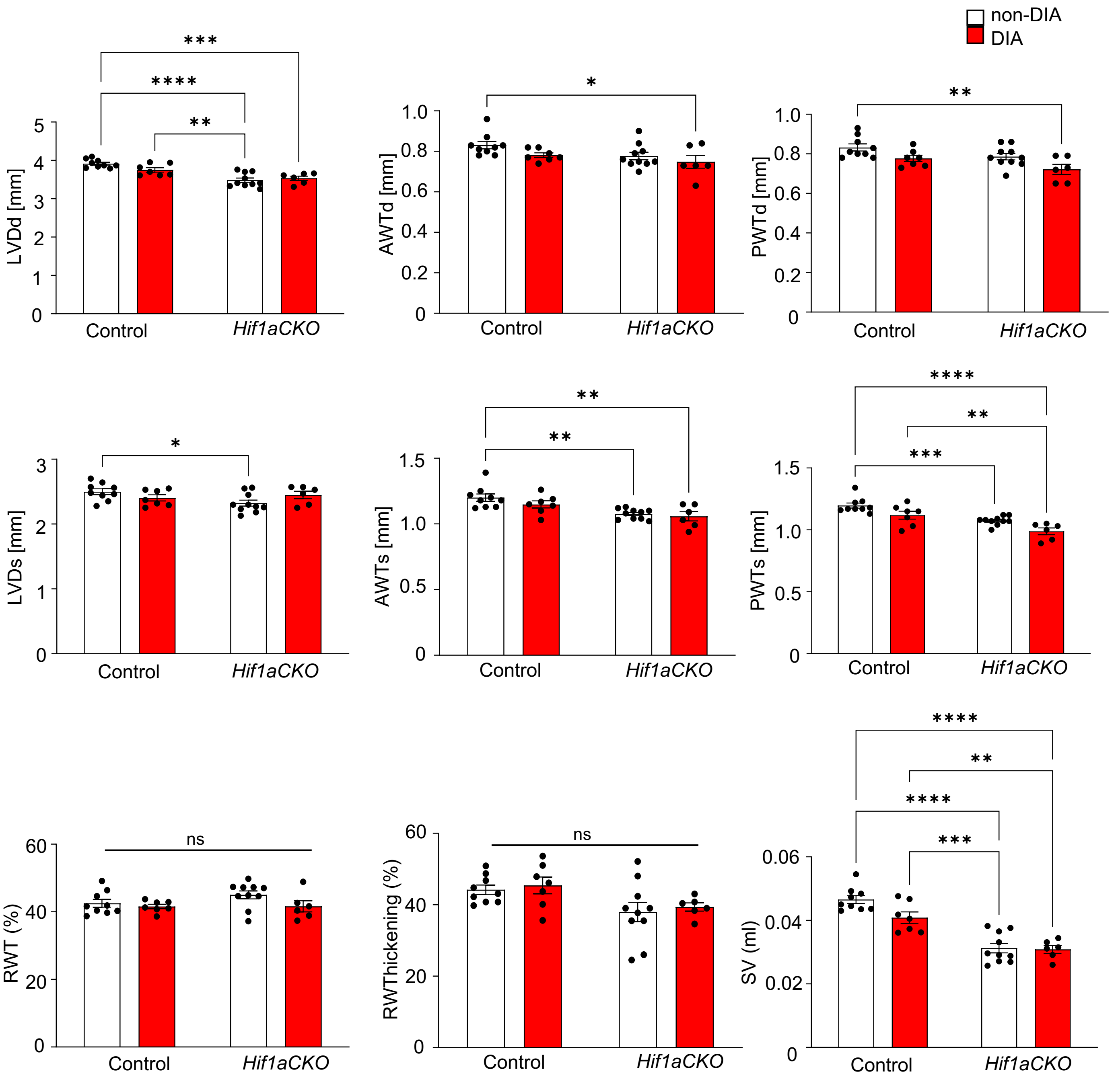

Figure S2. Basal left ventricular echocardiographic parameters

The echocardiographic evaluation of left ventricular systolic function of non-diabetic and diabetic control and *Hif1aCKO* mice (non-DIA Control n = 9, DIA Control n = 7, non-DIA *Hif1aCKO* n = 10, DIA *Hif1aCKO* n = 6). Two-Way ANOVA followed by Tukey's comparison multiple tests were used. Data are mean ± SEM; *$P < 0.05$, **$P < 0.01$, ***$P < 0.001$, **** $P < 0.0001$; ns = non-significant. Abbreviations: AWTd, diastolic anterior wall thickness; AWTs, systolic anterior wall thickness; LV, left ventricle; LVDd, diastolic cavity diameter; LVDs, systolic cavity diameter; PWTd, diastolic posterior wall thickness; PWTs, systolic posterior wall thickness; RWT, relative wall thickness; SV, stroke volume.

Legends for Videos

Video S1. Non-diabetic control stellate ganglion. Microdissected stellate ganglion of tdTomato reporter control-*Ai14* mice was cleared (CUBIC protocol), imaged, and reconstructed in 3D using light-sheet fluorescence microscopy (LFSM). Video shows the distribution tdTomato$^+$ sympathetic neurons in the anatomical microenvironment of the ganglion.

Video S2. Diabetic control stellate ganglion. Microdissected stellate ganglion of diabetic tdTomato reporter control-*Ai14* mice was cleared (CUBIC protocol), imaged, and reconstructed in 3D using light-sheet fluorescence microscopy (LFSM). Video shows the distribution tdTomato$^+$ sympathetic neurons in the anatomical microenvironment of the ganglion.

Video S3. Non-diabetic *Hif1aCKO* stellate ganglion. Microdissected stellate ganglion of tdTomato reporter *Hif1aCKO-Ai14* mice was cleared (CUBIC protocol), imaged, and reconstructed in 3D using light-sheet fluorescence microscopy (LFSM). Video shows the distribution tdTomato$^+$ sympathetic neurons in the anatomical microenvironment of the ganglion.

Video S4. Diabetic *Hif1aCKO* stellate ganglion. Microdissected stellate ganglion of diabetic tdTomato reporter *Hif1aCKO-Ai14* mice was cleared (CUBIC protocol), imaged, and reconstructed in 3D using light-sheet fluorescence microscopy (LFSM). Video shows the distribution tdTomato$^+$ sympathetic neurons in the anatomical microenvironment of the ganglion.

Video S5. The secondary sympathetic chain of non-diabetic Control. Microdissected the stellate and four upper ganglia of the thoracic sympathetic chain of tdTomato reporter control-*Ai14* mice were cleared (CUBIC protocol), imaged, and reconstructed in 3D using light-sheet fluorescence microscopy (LFSM). Video shows the distribution tdTomato$^+$ sympathetic neurons in the anatomical microenvironment of the ganglion.

Video S6. The secondary sympathetic chain of diabetic Control. Microdissected the stellate and four upper ganglia of the thoracic sympathetic chain of diabetic tdTomato reporter control-*Ai14* mice were cleared (CUBIC protocol), imaged, and reconstructed in 3D using light-sheet fluorescence microscopy (LFSM). Video shows the distribution tdTomato$^+$ sympathetic neurons in the anatomical microenvironment of the ganglion.

Video S7. The secondary sympathetic chain of non-diabetic *Hif1aCKO*. Microdissected the stellate and four upper ganglia of the thoracic sympathetic chain of tdTomato reporter *Hif1aCKO-Ai14* mice were cleared (CUBIC protocol), imaged, and reconstructed in 3D using light-sheet fluorescence microscopy (LFSM). Video shows the distribution tdTomato$^+$ sympathetic neurons in the anatomical microenvironment of the ganglion.

Video S8. The secondary sympathetic chain of diabetic *Hif1aCKO*. Microdissected the stellate and four upper ganglia of the thoracic sympathetic chain of diabetic tdTomato reporter *Hif1aCKO-Ai14* mice were cleared (CUBIC protocol), imaged, and reconstructed in 3D using light-sheet fluorescence microscopy (LFSM). Video shows the distribution tdTomato$^+$ sympathetic neurons in the anatomical microenvironment of the ganglion.
